# Frailty in Hepatocellular Carcinoma: An Unsettled Clinical Challenge

**DOI:** 10.3390/curroncol33010058

**Published:** 2026-01-19

**Authors:** Antonio Bonato, Pietro Guerra, Alessandro Vitale, Andrea Martini

**Affiliations:** 1 Unit of Internal Medicine and Hepatology (UIMH), Department of Medicine (DIMED), University of Padova, 35128 Padova, Italy; antonio.bonato@studenti.unipd.it (A.B.); pietro.guerra@studenti.unipd.it (P.G.); 2 General Surgery 2, Hepato-Pancreato-Biliary Surgery and Liver Transplantation, University of Padova, 35128 Padova, Italy; alessandro.vitale@unipd.it; 3 Unit of Internal Medicine and Hepatology (UIMH), Department of Medicine (DIMED), Azienda Ospedale Università Padova, 35128 Padova, Italy

**Keywords:** frailty, cirrhosis, hepatocellular carcinoma

## Abstract

Frailty is a clinical syndrome originally described in geriatrics but increasingly recognized across multiple medical fields. A wide variety of clinical tools have been developed to identify and quantify frailty in different contexts. In oncology, the Performance Status (PS) has long guided therapeutic decisions; however, with the evolution of cancer treatments and the aging of the patient population, a more comprehensive assessment of frailty is emerging as a valuable clinical tool. In patients with cirrhosis, frailty may manifest earlier than in the general population, and the Liver Frailty Index (LFI) has gained prominence as a validated measure among liver transplant candidates. Individuals with hepatocellular carcinoma (HCC) may exhibit frailty due to both the underlying cirrhosis and tumor burden. Nonetheless, evidence on the role of frailty in guiding treatment decisions for HCC remains limited, and standardized assessment tools are still lacking to optimize patient stratification and therapeutic allocation.

## Simple Summary

Frailty describes a state of reduced physiological reserve that increases vulnerability to adverse health outcomes. Although traditionally associated with older adults, frailty is increasingly recognized in younger patients with chronic diseases, including those with hepatocellular carcinoma (HCC). Patients with HCC often have underlying liver cirrhosis, which may contribute to frailty independently of age and tumor burden. This review summarizes current evidence on frailty in HCC, highlighting available assessment tools, their clinical relevance, and existing knowledge gaps. Understanding and measuring frailty in this population may improve prognostic stratification and support more individualized treatment decisions, although standardized approaches and dedicated studies are still lacking.

## 1. Introduction

**Figure 1:**
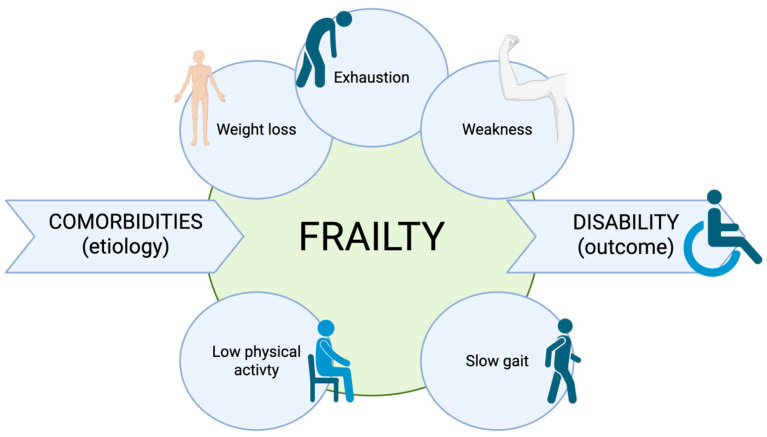
Representative definition of frailty according to current literature.

The concept of frailty was originally introduced in geriatrics. According to the Fried frailty phenotype definition [1], frailty is a clinical syndrome, more common as people age, characterized by weight loss, exhaustion, weakness, slow gait speed, and low physical activity. This condition of decreased reserve and resistance to stressors reflects a state of high vulnerability for adverse health outcomes, including disability, dependency, falls, long-term care needs, and mortality. Frailty should therefore be regarded as distinct from disability, which represents an outcome of the syndrome, and from comorbidity, which constitutes an etiological risk factor [1,2]. Figure 1 provides a representation of the concept of frailty.

In recent years, the concept of frailty has gained increasing recognition, extending beyond geriatrics into other fields including hepatology, oncology and surgery. Multiple frailty assessment tools have been developed and validated across various clinical contexts. Useful guides with an overview of frailty measurement instruments, including calculators, can be found online [3].

Identifying frail patients is particularly valuable for predicting clinical outcomes and risk, thereby guiding management decisions in the context of stressful treatments such as surgery or systemic therapy. However, frailty should not serve as a convenient justification for withholding potentially effective therapies, but rather as an opportunity to promote truly patient-centered care. This also means to increase physiological reserve and to prevent stressors (such as inappropriate medications) [4].

## 2. Cancer and Frailty

Frailty is gaining increasing attention in the field of oncology; however, the most widely used clinical tool for assessing decline in physical performance remains the Performance Status (PS) scale.

Initially assessed using the Karnofsky Performance Status (KPS) [5] developed in 1949, and later simplified in 1982 into the Eastern Cooperative Oncology Group (ECOG) Performance Status [6], the PS remains in use today for the design of oncology clinical trials and as a triage tool for therapeutic decision-making.

Despite its widespread and longstanding use, the limitations of the PS have become an issue with the evolution of oncology. The PS is a purely subjective assessment tool, originally developed in an era when chemotherapy represented the only available systemic treatment, whereas today therapeutic strategies include Immune checkpoint inhibitors (ICIs) and tyrosine kinase inhibitors (TKIs), and the population age and characteristics are changing too. Moreover, PS captures only tumor-related burden, potentially assigning the same score to patients with markedly different baseline health statuses, one otherwise healthy and the other already impaired by comorbidities [7].

In this context, increasing attention is being directed toward the concept of frailty, mainly in the elderly population, which offers a more comprehensive assessment of the patients and their overall functional reserve. Both cancer and oncologic treatments are potential significant stressors to challenge physiological reserve in frail patients. Frailty is highly prevalent among older cancer patients and is associated with an increased risk of all-cause mortality, postoperative mortality, chemotherapy intolerance, and postoperative complications [8,9].

In older adults, undergoing a comprehensive geriatric assessment alongside systemic therapy—allowing the identification of medical, social, and functional needs to guide personalized management—has been shown to improve quality of life and optimize health-care delivery in patients receiving systemic anticancer treatment [10].

Thus, most oncologic studies on frailty focus on older adults; however, it is important to recognize that younger patients may also be frail, as cancer itself can contribute to the decline of physiological reserve.

## 3. Cirrhosis and Frailty

Older adults exhibit two primary pathways leading to the development of frailty: one mediated by physiological aging processes, such as sarcopenia and anorexia [11,12], and another driven by the presence of severe disease or multiple comorbidities [1]. In patients with cirrhosis, the presence of severe underlying conditions is evident; furthermore, sarcopenia, a hallmark of advanced liver disease [13,14], emerges well before the geriatric age and is associated with a higher risk of mortality [14]. Consequently, extending the concept of frailty to the cirrhotic population appears both logical and clinically relevant.

Frailty and sarcopenia are two conditions that have been shown to be highly prevalent in the cirrhotic patient population, with frailty affecting 18–43% of clinically stable cirrhosis [15]. The two concepts appear to be closely related, as sarcopenia reflects impaired muscle mass, while physical frailty denotes impaired muscle function. However, studies evaluating both parameters in liver transplant candidates have demonstrated a low correlation between the two conditions [16,17,18], suggesting that they may in fact represent distinct clinical domains.

Indeed, frailty results from dysfunction in musculoskeletal and a broad range of physiological systems, including the cardiovascular, neurological, endocrine, and immune systems; all of which may be adversely affected in the setting of cirrhosis [19].

Although the biological mechanisms underlying the development of frailty remain incompletely understood, several contributing factors have been identified in older adults; many of which can affect patients with cirrhosis even at a younger age: among them chronic inflammation [20,21], deregulated nutrient sensing and hormonal changes [4].

Initial studies employing various frailty assessment scales in the cirrhotic population have demonstrated that frailty is associated with increased mortality, higher rates of decompensation and hospitalization among individuals on the liver transplant waitlist [22,23], increased mortality and hospitalization also among those with non-advanced cirrhosis [24,25,26,27,28] and increased mortality for inpatients admitted for decompensated cirrhosis [29,30].

The concept, therefore, is that frailty may offer additional prognostic value beyond traditional scoring systems such as MELD and Child-Pugh, by quantifying aspects of a patient’s clinical condition that are commonly evaluated by general clinical assessment but are often limited by subjectivity. Such an aspect that has been shown to correlate with frailty in cirrhotic patients is the presence of depressive symptoms that are major determinants of Health-Related Quality of Life (HRQL) [31].

As early as the original definition of the frailty phenotype, Fried et al. demonstrated that greater depressive symptomatology was associated with frailty; notably, the assessment of poor endurance and energy incorporated items from the CES-D scale, originally developed to evaluate depressive symptoms [1]. Cron et al., in a study involving patients with end-stage liver disease referred for liver transplant evaluation, found that depression was a common condition in this population and that frailty was strongly associated with this condition, whereas MELD score showed no such association [32].

HRQL is impaired even in patients with uncomplicated forms of cirrhosis, primarily due to fatigue [33,34] and “minor symptoms” such as muscular cramps and pruritus that are frequently underrecognized by physicians. Also, depression has been demonstrated to be a major determinant of the HRQL [35,36].

As frailty affects patients with cirrhosis before the geriatric age, a similar anticipatory pattern is observed with respect to HRQL. Furthermore, the impact of cirrhosis on HRQL is strongly age-dependent, with the greatest deviation from population norms observed in the youngest patients [37].

Frailty has been proven to be reversible in the elderly through nutritional, physical and cognitive interventions [38,39]. Cirrhosis is a catabolic state in which nutritional interventions and physical exercise are recommended to avoid sarcopenia and lower the risk of decompensation or facilitate the recovery [40]. However, no specific studies have yet evaluated targeted interventions for frailty in the cirrhotic population.

### 3.1. Assessment of Frailty in Cirrhosis

Dozens of tools have been proposed to measure frailty; the more commonly used in cirrhotic patients are shown in Table 1.

Others performance-based tools used are gait speed alone [22], handgrip strength alone [17] and the 6 min-walk-test [16]. All these scores were compared in an Indian cohort and appeared to be equivalent in identifying frailty in patients with cirrhosis, as well as in predicting six-month mortality and hospitalization [25].

### 3.2. The Liver Frailty Index

The only tool specifically validated in the cirrhotic population is the Liver Frailty Index (LFI). In 2017, Lai et al. developed an index to measure frailty, evaluating frailty measurement-tools already used in the geriatric population: four performance-based tools (gait speed, grip strength, chair stands, and balance) and five self-reported tools (unintentional weight loss, exhaustion, physical activity, ADL, and iADL). LFI was then validated in a non-geriatric cirrhotic population waiting for liver-transplantation; it assesses grip strength, chair stands, and balance. This index, which stratifies patients as “robust,” “pre-frail,” and “frail,” associated with MELD-Na improves risk prediction of transplant waitlist mortality over MELD-Na alone [23]. Later, a telemedicine version was developed: the Tele-Liver Frailty Index (TeLeFI) [43]. Like other scores, the LFI identifies a pre-frail state, characterized by individuals who are not yet frail but at increased risk of progressing to frailty.

Interestingly, compared to pre-transplant values, frailty worsens at 3 months post-LT and shows only modest improvement by 12 months, with just one-third of patients achieving robustness. Notably, pretransplant LFI was a strong predictor of post-transplant recovery of robustness [44].

The LFI does not incorporate subjective components in its evaluation, but the addition of the LFI to clinician-based subjective assessment significantly improved the prediction of waitlist mortality compared to subjective assessment alone [45]. Today, the LFI, although validated in the liver transplant waitlist population, represents the most widely adopted and clinically utilized instrument for frailty assessment in patients with cirrhosis, and has been demonstrated to predict mortality and hospitalization beyond the liver transplant waitlist population [25].

The LFI has also been compared with the Karnofsky Performance Status (KPS) that is routinely used in patients assessment for LT waitlist admission in the US, showing a stronger association with waitlist mortality than KPS [46]. Worsening LFI has also been associated with an increased risk of mortality and waitlist dropout [47] and frail LT recipients are at increased risk of post-transplant mortality, worse global functional health (i.e., disability, quality of life) and have higher post-transplant healthcare utilization [48,49].

As a multidimensional syndrome, general frailty encompasses not only physical but also cognitive, emotional, and psychosocial domains. The LFI, however, focuses exclusively on physical impairment. This may represent a limitation, further compounded by the absence of self-reported components and the exclusion of gait speed, which, among physical performance tests, has demonstrated the highest sensitivity in identifying frailty as defined by the Fried phenotype [50]. Moreover, as a performance-based assessment, the LFI cannot be reliably administered in acute care settings, such as during hospitalization.

It should be noted, however, that the LFI was specifically validated in cirrhotic, rather than geriatric, populations, and in most of Lai’s studies on the LFI, patients with HCC were not excluded, except for a few instances [45].

## 4. Hepatocellular Carcinoma and Frailty

Primary liver cancer is the sixth most common cancer globally and the third leading cause of cancer-related death. Hepatocellular carcinoma (HCC) is the predominant liver cancer, accounting for approximately 90% of cases; its incidence and mortality are two to three times higher in men. The primary risk factor for the development of HCC is the presence of cirrhosis, which is found in over 80% of patients. Consequently, other risk factors for HCC are those that lead to the development of cirrhosis, and their relative contribution varies geographically. Chronic HBV and HCV infection remain the leading causes worldwide, followed by alcohol-related liver disease and the emerging metabolic dysfunction-associated steatotic liver disease (MASLD) [51,52].

The main clinical prognostic factors in patients with HCC are tumor burden, liver function, and overall HCC-related health status. The cancer staging system that currently provides prognostic information and guides management and treatment decisions in HCC is the Barcelona Clinic Liver Cancer (BCLC) classification [53]. According to BCLC, HCC-related general health status is evaluated with the ECOG-PS and it is determinant in defining the stage of the disease. It incorporates tumor-related symptoms that were not present prior to cancer diagnosis, but in cirrhotic patients it can be difficult to differentiate between tumor and liver-dysfunction symptoms adding complexity to clinical management.

Patients with HCC, therefore, have a baseline elevated risk of being frail due to the underlying cirrhosis. As a result, it becomes clinically relevant to explore how frailty can be assessed in this specific population to improve prognostic stratification and guide more appropriate and individualized clinical management, also in younger patients.

There is little evidence on the role of frailty on prognosis and on treatment allocation in patients with HCC and currently, no clinical guideline recommends the routine assessment of frailty in patients with HCC. In a large retrospective study, Ramai et al. demonstrated that frailty in hospitalized patients with HCC, as measured by the Hospital Frailty Risk Score (HFRS)—a tool based on the presence of comorbidities—was associated with an increased risk of mortality and hepatic encephalopathy, as well as longer hospital stays and higher hospitalization costs [54].

Sarcopenia is highly prevalent and represents a prognostic factor in cirrhosis and HCC [55]; moreover, it has been associated with increased rates of major complications in patients with advanced cirrhosis undergoing liver resection for HCC [56]. Hirota et al. investigated the correlation between the Liver Frailty Index (LFI) and muscle atrophy. They found that pre-frail/frail LFI was an independent factor associated with muscle atrophy in patients with cirrhosis and HCC, and that the LFI could predict muscle atrophy with good sensitivity even in patients with normal grip strength, which is traditionally used to assess the “sarcopenia component” of frailty [57].

### 4.1. The Multiparametric Management of HCC

In this context, the concept of a multiparametric therapeutic hierarchy has been proposed, in which treatment options are ranked according to their expected survival benefit, and patients are stratified as fit or unfit based on general conditions rather than solely on cancer-related symptoms. Complementary to this, the notion of a converse therapeutic hierarchy has been introduced, whereby therapies are prioritized not for their immediate impact on survival, but for their potential to enable or optimize subsequent curative treatments [58]. The concept of fitness was introduced to overcome the limitations of Performance Status (PS) in evaluating a patient’s overall condition. It can be defined as the ability to tolerate a given treatment based on biological, physical, and clinical factors, and is primarily determined by age, frailty, and comorbidities [59]. Therefore, within the multiparametric assessment, frailty represents a key component of fitness. Both fitness and frailty should be regarded as dynamic concepts, potentially modifiable through targeted interventions. Future research is needed to develop tailored strategies for patients with different fitness profiles, aiming to optimize treatment outcomes and minimize treatment-related adverse effects [60].

### 4.2. HCC Treatment and Frailty, Actual Evidence

Only a limited number of studies have evaluated frailty in the context of HCC therapies, these and others studies about frailty and HCC are summarized in Table 2. All these studies employ different frailty assessment tools, each capturing distinct dimensions of the syndrome. Some require objective measurements or performance-based evaluations, while others rely on subjective or patient-reported assessments. For this reason, combined with the relatively small number of studies, meaningful comparisons across studies are precluded, and the development of actionable clinical guidance remains limited at present.

#### 4.2.1. Surgery and Locoregional Therapies

The surgical options for HCC with curative intent are liver resection and liver transplantation (LT). Eligibility for surgery requires a multiparametric assessment, including liver function, portal hypertension, remnant liver volume and function, as well as prediction of early postoperative outcomes. Importantly, age should not be considered an absolute contraindication to surgery [51].

Frailty correlates with mortality and morbidity across surgical specialties [70], including major abdominal surgery [71]. Moreover, incorporating frailty screening into the preoperative evaluation and integrating it into surgical decision-making has been associated with reduced mortality [72].

A variety of locoregional therapies (LRT) are also available, including percutaneous ablation, intra-arterial embolization techniques, and external beam radiation therapy. LRT may serve as an alternative to surgery to achieve complete tumor ablation, but they also play a central role in the management of more advanced stages of the disease [51,52].

Patients awaiting LT are becoming older and more comorbid, while cirrhosis itself accelerates physiological aging beyond chronological age [15]. The prevalence of physical frailty among LT candidates is higher in those over 65; however, its adverse impact on waitlist outcomes appears similar between younger and older patients, corresponding to nearly a twofold increased risk of waitlist mortality [73]. LT candidates with HCC are generally older than those without HCC and may therefore carry a higher burden of non-hepatic comorbidities contributing to frailty. However, they often present with less advanced synthetic dysfunction and fewer portal hypertensive complications [51]. Frailty and sarcopenia are highly prevalent among patients listed for liver transplantation, regardless of the presence of HCC. Frailty appears to be a stronger predictor of mortality, mostly in non-HCC patients, probably reflecting the greater impact of liver decompensation compared to the cancer-related component of frailty [18]. DeMaria et al. investigated the relationship between frailty and waitlist outcomes in HCC patients listed for liver transplantation. They found that frailty was associated with longer post-transplant hospital stay, without significant differences in ICU stay or 30-day mortality [61].

Recently, Jutras et al. studied a large cohort of HCC LT candidates and found that tumor burden, total tumor diameter, and AFP were not associated with frailty assessed with LFI. Since patients with HCC awaiting liver transplantation generally have well-compensated liver disease, these findings suggest that frailty in this setting may not be driven by liver failure or tumor burden [69].

Frailty assessed with Clinical Frailty Scale (CFS), in elderly patients with HCC undergoing surgical resection, proved to be a strong predictor of both short-term outcomes (30- and 90-day mortality) and long-term outcomes (overall survival and progression free survival) [62,66]. Frailty evaluated with Kihon Checklist (KCL), a self-administered list of 25 questions about different frailty domains, is associated with unfavorable long-term outcomes, overall survival (but not the non–disease-free survival), and a higher incidence of extrahepatic recurrence following liver resection in elderly patients with HCC [64].

In a retrospective study of a population with HCC on the liver transplant waitlist who underwent locoregional therapy (ablation or chemoembolization), frailty remained stable over time. These results suggest that LRT is well tolerated in patients awaiting liver transplantation [67]. Shao et al. were the first to apply the 5-factor Modified Frailty Index (mFI-5) to assess frailty in an elderly population undergoing transarterial chemoembolization (TACE) for HCC. They demonstrated that, in this population—often considered for locoregional treatment—frail patients exhibited reduced overall survival following TACE, whereas no significant difference was observed in progression-free survival. This frailty index is based solely on the presence of comorbidities and does not incorporate any physical performance testing [66].

#### 4.2.2. Systemic Therapies

Systemic therapy for HCC currently relies on immune checkpoint inhibitors (ICIs) and targeted therapies, while conventional cytotoxic agents have not shown clinical benefit. ICIs act mainly by blocking PD-1 or PD-L1 pathways or CTLA4, restoring antitumor immune response. Targeted therapies include tyrosine kinase inhibitors (TKIs) and agents that inhibit the VEGF signaling pathway, exerting an antiangiogenic effect. The benefit of systemic therapy has been demonstrated in patients with advanced-stage HCC or in those with disease progression after LRT. The combination of ICIs and TKIs alone currently represents the mainstay of systemic treatment [51,52,53].

In the generic oncological population, focusing on the elderly population, the introduction of immune checkpoint inhibitors (ICIs) has enabled the provision of effective therapies with improved tolerability. Consequently, an increasing proportion of older adults are being offered oncologic treatment, and the elderly more commonly present with frailty and multiple comorbidities. The incidence of severe adverse events associated with ICIs does not appear to differ significantly between older and younger populations; however, low-grade adverse events, often leading to treatment discontinuation, seem to occur more frequently in older patients [74]. Interestingly, in patients with metastatic non–small cell lung cancer (NSCLC) treated with pembrolizumab, frailty rather than age has been shown to be associated with the development of ICI-related adverse events [75], as well as with overall survival and progression-free survival [76].

At present, except for the study by Ozluk et al., which demonstrated that frailty is associated with worse overall survival in patients with HCC undergoing LRT or ICI treatment [65], no studies have specifically investigated the role of frailty in HCC patients receiving immunotherapy. This study score frailty using the CARE-FI, a 44-item tool validated for older adults with gastrointestinal malignancy that utilizes a deficit accumulation model to quantify frailty, for example, activities of daily living, history of falls, and comorbidity.

### 4.3. Frailty-Targeted Interventions

Targeted interventions, such as physical exercise and oral nutritional supplementation, have been proposed to improve frailty in older adults, in whom frailty has been shown to be at least partially reversible. However, evidence regarding the effectiveness and cost-effectiveness of such interventions remains limited [4]. Similarly, in patients with cirrhosis—where frailty is highly prevalent—interventional data are scarce and largely heterogeneous, highlighting an important unmet clinical need [31]. 2021 practice guidance from the AASLD [77] highlights the importance of targeted nutritional support and structured physical activity as part of the integrated management of malnutrition, frailty, and sarcopenia in cirrhosis. Nutritional optimization, including personalized caloric and protein intake and avoidance of prolonged fasting, along with progressive exercise interventions, can mitigate muscle wasting and functional decline. Regular reassessment and multidisciplinary care are recommended to tailor and monitor these interventions.

Oncology is increasingly adopting rehabilitation assessments and interventions to address physical and cognitive impairments and to manage treatment-related symptoms across different cancer types. Accordingly, several guidelines have been developed to guide resource allocation, and research on physical activity now spans multiple phases of the cancer continuum, from prevention and treatment to rehabilitation, palliation, and survivorship [78,79]. Similarly, in the field of abdominal surgery, prehabilitation strategies have shown promising potential in improving postoperative outcomes [60,80].

Although evidence on interventions targeting frailty in patients with HCC is lacking, a noteworthy study by Tsuchihashi et al. demonstrated that in-hospital exercise programs improved frailty, as measured by the Liver Frailty Index (LFI) [63].

## 5. Future Perspectives and Limitations

The assessment of frailty is gaining increasing clinical relevance and is now applied across hepatology, oncology, and surgery. However, the definition of frailty itself remains broad, and the different tools proposed capture distinct domains such as physical performance, comorbidities, symptoms, polypharmacy, social support, and nutritional status. As a result, hundreds of tools have been developed both in geriatric and non-geriatric settings, many of which overlap but often measure different aspects of vulnerability.

To facilitate both the understanding and the practical use of frailty, greater uniformity in its measurement within each clinical field would be desirable, as this would also allow more robust comparison of data across studies. In oncology—including HCC—the Karnofsky Performance Status and the ECOG Performance Status are still widely used. Nevertheless, these scales were developed decades ago in the era of chemotherapy, and they may not adequately reflect the reality of modern treatments such as immunotherapy. In this setting, frailty tools may have significant potential to personalize both surgical and systemic management.

To date, studies on frailty in HCC remain limited, and even fewer have examined its relationship with specific therapeutic approaches. Since most patients with HCC have underlying cirrhosis, it is important to disentangle the contribution of liver disease from that of the tumor itself. Some evidence suggests that in HCC candidates for liver transplantation—who often present with compensated disease and preserved liver function—frailty may not be directly associated with tumor burden or characteristics. Conversely, in other HCC populations undergoing alternative treatments, different patterns may emerge.

Currently, the Liver Frailty Index (LFI) is the only frailty score specifically validated in patients with cirrhosis and, therefore, holds promise for broader application in the HCC population. However, only a limited number of studies have evaluated the LFI specifically in patients with HCC, underscoring the need for further research in this area.

In conclusion, further studies are needed to validate the most appropriate tool for assessing frailty in patients with cirrhosis and hepatocellular carcinoma, and to determine its true impact on patient prognosis. Once reliable assessment methods are established, future research should also focus on identifying effective interventions to modify or reverse frailty itself.

## Figures and Tables

**Table 1:** Frailty assessment tools commonly used in cirrhosis.

Frailty Tool	Description	Frailty Threshold
Fried Frailty Scale (FFS) [1]	Self-report and performance-based, evaluates 5 criteria: weight loss, exhaustion, leisure time activity level, gait speed and grip strength	3 or more criteria
Clinical Frailty Scale (CSF) [24]	Based on clinical judgment, define 9 categories from “very fit” to “terminally ill”	CFS > 4
Short Physical Performance Battery (SPPB) [41]	Performance-based, evaluates balance, gait speed and chair stands; each component is individually scored, to a total score from 0 to 12	SPPB < 10
Liver Frailty Index (LFI) [23]	Performance-based, evaluates handgrip strength, balance and chair stands. It gives a score using an online calculator [42]	LFI > 4.5

**Table 2:** Studies about frailty in HCC patients.

Reference	Frailty Tool	Objective vs. Subjective Tool	Design of Study	Population	Impact
DeMaria et al., 2019 [61]	FFS	Subjective	Single center, prospective observational	50 HCC patients (median age 64) admitted into LT waitlist	Frailty is associated with longer hospital stay in HCC patients who underwent LT
Hirota et al., 2020 [57]	LFI	Objective	Single center, prospective observational	138 elderly (over 70) HCC cirrhotic patients, hospitalized	LFI predicted muscle atrophy with high sensitivity, even in patients with normal grip strength
Yamada et al., 2021 [62]	CFS	Subjective	Single center, prospective observational	92 elderly (over 75) HCC patient undergoing hepatectomy	Frailty can estimate the prognosis of HCC patients who underwent hepatectomy (frail group showed worst OS and PFS)
Ramai et al., 2021 [54]	HFRS	Comorbidity based	Multi center, retrospective observational	10,983 HCC patients (median age 62)	Frailty is an independent predictor of encephalopathy and in-patient mortality in HCC patients
Tsuchihashi et al., 2021 [63]	LFI	Objective	Multi center, prospective observational	181 HCC patients (median age 77)	In-hospital exercise improved frailty in HCC patients
Okada et al., 2024 [64]	KCL	Subjective	Single center, prospective observational	81 elderly (over 65) patients undergoing hepatic resection	Frailty is associated with unfavorable long-term outcomes after liver resection in elderly HCC patients
Ozluk et al., 2024 [65]	CARE-FI	Mixed	Single center, prospective observational	166 elderly (over 60) HCC patients	Being frail is associated with worse OS in patients with HCC (LRT or ICI treatment)
D’Arcangelo et al., 2024 [18]	LFI	Objective	Single center, prospective observational	105 patients (42 with HCC, median age 65) admitted into LT waitlist	Frail patients, especially without HCC, tend to have worse outcomes and to be more decompensated, no difference in age among frail and non-frail
Diao et al., 2024 [66]	CFS	Subjective	Multi center, prospective observational	488 elderly (over 70) HCC patients undergoing hepatic resection	Frailty is significantly associated with adverse short-term and long-term outcomes (OS and PFS) after resection in elderly patients with HCC
Hu et al., 2025 [67]	LFI	Objective	Single center, retrospective observational	86 HCC patients (median age 62) undergoing LRT	LFI remained stable over time after LRT, suggesting that LRT is generally well tolerated awaiting LT
Shao et al., 2025 [68]	mFI-5	Comorbidity based	Single center, retrospective observational	143 elderly (over 65) HCC patients undergoing TACE	The mFI-5 is a useful predictor of long-term OS (not FPS) in elderly HCC patients treated with TACE
Jutras et al., 2025 [69]	LFI	Objective	Single center, retrospective observational	2420 patients (801 with HCC) admitted into LT waitlist	Frailty is associated with hepatic and non-hepatic factors, but not with tumor characteristics

CARE-FI, CARE frailty index (personalized for this study) CFS, clinical frailty scale; FFS, fried frailty scale; HCC, hepatocellular carcinoma; HFRS, hospital frailty risk score; ICI, immune checkpoint inhibitors; KCL, Kihon checklist; LFI, liver frailty index; LRT, locoregional treatment; LT, liver transplantation; mFI-5, 5-factor modified frailty index; OS, overall survival; PFS, progression free survival; TACE, transarterial chemoembolization.

## Data Availability

No new data were created or analyzed in this study. Data sharing is not applicable to this article.

## Abbreviations

The following abbreviations are used in this manuscript:

HCC	Hepatocellular carcinoma
PS	Performance Status
HRQL	Health related quality of life
LFI	Liver Frailty Index
BCLC	Barcelona Clinic Liver Cancer
LT	Liver transplantation
LRT	Locoregional treatment
ICI	Immune checkpoint inhibitor
TKI	Tyrosine kinase inhibitor

## References

[1] Fried L.P., Tangen C.M., Walston J., Newman A.B., Hirsch C., Gottdiener J., Seeman T., Tracy R., Kop W.J., Burke G. (2001). Frailty in Older Adults: Evidence for a Phenotype. J. Gerontol. Ser. A.

[2] Fried L.P., Ferrucci L., Darer J., Williamson J.D., Anderson G. (2004). Untangling the Concepts of Disability, Frailty, and Comorbidity: Implications for Improved Targeting and Care. J. Gerontol. Ser. A Biol. Sci. Med. Sci..

[3] eFRAILTY. https://efrailty.hsl.harvard.edu.

[4] Kim D.H., Rockwood K. (2024). Frailty in Older Adults. N. Engl. J. Med..

[5] Schag C.C., Heinrich R.L., Ganz P.A. (1984). Karnofsky Performance Status Revisited: Reliability, Validity, and Guidelines. J. Clin. Oncol. Off. J. Am. Soc. Clin. Oncol..

[6] Oken M.M., Creech R.H., Tormey D.C., Horton J., Davis T.E., McFadden E.T., Carbone P.P. (1982). Toxicity and Response Criteria of the Eastern Cooperative Oncology Group. Am. J. Clin. Oncol..

[7] Simcock R., Wright J. (2020). Beyond Performance Status. Clin. Oncol..

[8] Handforth C., Clegg A., Young C., Simpkins S., Seymour M.T., Selby P.J., Young J. (2015). The Prevalence and Outcomes of Frailty in Older Cancer Patients: A Systematic Review. Ann. Oncol..

[9] Ethun C.G., Bilen M.A., Jani A.B., Maithel S.K., Ogan K., Master V.A. (2017). Frailty and Cancer: Implications for Oncology Surgery, Medical Oncology, and Radiation Oncology. CA Cancer J. Clin..

[10] Soo W.K., King M.T., Pope A., Parente P., Dārziņš P., Davis I.D. (2022). Integrated Geriatric Assessment and Treatment Effectiveness (INTEGERATE) in Older People with Cancer Starting Systemic Anticancer Treatment in Australia: A Multicentre, Open-Label, Randomised Controlled Trial. Lancet Healthy Longev..

[11] Evans W.J. (1995). What Is Sarcopenia?. J. Gerontol. Ser. A Biol. Sci. Med. Sci..

[12] Je M. (1997). Anorexia of Aging: Physiologic and Pathologic. Am. J. Clin. Nutr..

[13] Ooi P.H., Hager A., Mazurak V.C., Dajani K., Bhargava R., Gilmour S.M., Mager D.R. (2019). Sarcopenia in Chronic Liver Disease: Impact on Outcomes. Liver Transplant. Off. Publ. Am. Assoc. Study Liver Dis. Int. Liver Transplant. Soc..

[14] Tantai X., Liu Y., Yeo Y.H., Praktiknjo M., Mauro E., Hamaguchi Y., Engelmann C., Zhang P., Jeong J.Y., van Vugt J.L.A. (2022). Effect of Sarcopenia on Survival in Patients with Cirrhosis: A Meta-Analysis. J. Hepatol..

[15] Tandon P., Zanetto A., Piano S., Heimbach J.K., Dasarathy S. (2023). Liver Transplantation in the Patient with Physical Frailty. J. Hepatol..

[16] Yadav A., Chang Y.-H., Carpenter S., Silva A.C., Rakela J., Aqel B.A., Byrne T.J., Douglas D.D., Vargas H.E., Carey E.J. (2015). Relationship between Sarcopenia, Six-Minute Walk Distance and Health-Related Quality of Life in Liver Transplant Candidates. Clin. Transplant..

[17] Sinclair M., Chapman B., Hoermann R., Angus P.W., Testro A., Scodellaro T., Gow P.J. (2019). Handgrip Strength Adds More Prognostic Value to the Model for End-Stage Liver Disease Score Than Imaging-Based Measures of Muscle Mass in Men with Cirrhosis. Liver Transplant. Off. Publ. Am. Assoc. Study Liver Dis. Int. Liver Transplant. Soc..

[18] D’Arcangelo F., Zanetto A., Ferrarese A., Gambato M., Lanari J., Piano S., Germani G., Senzolo M., Russo F.P., Angeli P. (2024). Frailty and Sarcopenia in Patients with Cirrhosis Awaiting Liver Transplantation: Evidence from a Single-Centre, Prospective Cohort Study. Updat. Surg..

[19] Tandon P., Montano-Loza A.J., Lai J.C., Dasarathy S., Merli M. (2021). Sarcopenia and Frailty in Decompensated Cirrhosis. J. Hepatol..

[20] Ferrucci L., Fabbri E. (2018). Inflammageing: Chronic Inflammation in Ageing, Cardiovascular Disease, and Frailty. Nat. Rev. Cardiol..

[21] Hubbard R.E., O’Mahony M.S., Savva G.M., Calver B.L., Woodhouse K.W. (2009). Inflammation and Frailty Measures in Older People. J. Cell. Mol. Med..

[22] Dunn M.A., Josbeno D.A., Tevar A.D., Rachakonda V., Ganesh S.R., Schmotzer A.R., Kallenborn E.A., Behari J., Landsittel D.P., DiMartini A.F. (2016). Frailty as Tested by Gait Speed Is an Independent Risk Factor for Cirrhosis Complications That Require Hospitalization. Am. J. Gastroenterol..

[23] Lai J.C., Covinsky K.E., Dodge J.L., Boscardin W.J., Segev D.L., Roberts J.P., Feng S. (2017). Development of a Novel Frailty Index to Predict Mortality in Patients with End-Stage Liver Disease. Hepatology.

[24] Tandon P., Tangri N., Thomas L., Zenith L., Shaikh T., Carbonneau M., Ma M., Bailey R.J., Jayakumar S., Burak K.W. (2016). A Rapid Bedside Screen to Predict Unplanned Hospitalization and Death in Outpatients with Cirrhosis: A Prospective Evaluation of the Clinical Frailty Scale. Off. J. Am. Coll. Gastroenterol. ACG.

[25] Singh S., Taneja S., Tandon P., Bansal A., Gorsi U., Roy A., De A., Verma N., Premkumar M., Duseja A. (2022). A Comparison of Different Frailty Scores and Impact of Frailty on Outcome in Patients with Cirrhosis. J. Clin. Exp. Hepatol..

[26] Deng Y., Lin L., Hou L., Fan X., Zhao T., Mao L., Wang X., Wang B., Ma Y., Sun C. (2020). A Self-Reported Frailty Index Predicts Long-Term Mortality in Hospitalized Patients with Cirrhosis. Ann. Transl. Med..

[27] Kremer W.M., Nagel M., Reuter M., Hilscher M., Michel M., Kaps L., Labenz J., Galle P.R., Sprinzl M.F., Wörns M.-A. (2020). Validation of the Clinical Frailty Scale for the Prediction of Mortality in Patients with Liver Cirrhosis. Clin. Transl. Gastroenterol..

[28] Wang S., Whitlock R., Xu C., Taneja S., Singh S., Abraldes J.G., Burak K.W., Bailey R.J., Lai J.C., Tandon P. (2022). Frailty Is Associated with Increased Risk of Cirrhosis Disease Progression and Death. Hepatology.

[29] Serper M., Tao S.Y., Kent D.S., Garren P., Burdzy A.E., Lai J.C., Gougol A., Bloomer P.M., Reddy K.R., Dunn M.A. (2021). Inpatient Frailty Assessment Is Feasible and Predicts Nonhome Discharge and Mortality in Decompensated Cirrhosis. Liver Transpl..

[30] Tapper E.B., Finkelstein D., Mittleman M.A., Piatkowski G., Lai M. (2015). Standard Assessments of Frailty Are Validated Predictors of Mortality in Hospitalized Patients with Cirrhosis. Hepatology.

[31] Laube R., Wang H., Park L., Heyman J.K., Vidot H., Majumdar A., Strasser S.I., McCaughan G.W., Liu K. (2018). Frailty in Advanced Liver Disease. Liver Int. Off. J. Int. Assoc. Study Liver.

[32] Cron D.C., Friedman J.F., Winder G.S., Thelen A.E., Derck J.E., Fakhoury J.W., Gerebics A.D., Englesbe M.J., Sonnenday C.J. (2016). Depression and Frailty in Patients with End-Stage Liver Disease Referred for Transplant Evaluation. Am. J. Transplant. Off. J. Am. Soc. Transplant. Am. Soc. Transpl. Surg..

[33] Younossi Z.M., Kremer A.E., Swain M.G., Jones D., Bowlus C., Trauner M., Henry L., Gerber L. (2024). Assessment of Fatigue and Its Impact in Chronic Liver Disease. J. Hepatol..

[34] Tapper E.B., Baki J., Parikh N.D., Lok A.S. (2019). Frailty, Psychoactive Medications, and Cognitive Dysfunction Are Associated with Poor Patient-Reported Outcomes in Cirrhosis. Hepatology.

[35] Nardelli S., Pentassuglio I., Pasquale C., Ridola L., Moscucci F., Merli M., Mina C., Marianetti M., Fratino M., Izzo C. (2013). Depression, Anxiety and Alexithymia Symptoms Are Major Determinants of Health Related Quality of Life (HRQoL) in Cirrhotic Patients. Metab. Brain Dis..

[36] Gazineo D., Godino L., Bui V., El Mouttaqi L., Franciosi E., Natalino A., Ceci G., Ambrosi E. (2021). Health-Related Quality of Life in Outpatients with Chronic Liver Disease: A Cross-Sectional Study. BMC Gastroenterol..

[37] Marchesini G., Bianchi G., Amodio P., Salerno F., Merli M., Panella C., Loguercio C., Apolone G., Niero M., Abbiati R. (2001). Factors Associated with Poor Health-Related Quality of Life of Patients with Cirrhosis. Gastroenterology.

[38] Tarazona-Santabalbina F.J., Gómez-Cabrera M.C., Pérez-Ros P., Martínez-Arnau F.M., Cabo H., Tsaparas K., Salvador-Pascual A., Rodriguez-Mañas L., Viña J. (2016). A Multicomponent Exercise Intervention That Reverses Frailty and Improves Cognition, Emotion, and Social Networking in the Community-Dwelling Frail Elderly: A Randomized Clinical Trial. J. Am. Med. Dir. Assoc..

[39] Ng T.P., Feng L., Nyunt M.S.Z., Feng L., Niti M., Tan B.Y., Chan G., Khoo S.A., Chan S.M., Yap P. (2015). Nutritional, Physical, Cognitive, and Combination Interventions and Frailty Reversal Among Older Adults: A Randomized Controlled Trial. Am. J. Med..

[40] European Association for the Study of the Liver (2019). EASL Clinical Practice Guidelines on Nutrition in Chronic Liver Disease. J. Hepatol..

[41] Guralnik J.M., Simonsick E.M., Ferrucci L., Glynn R.J., Berkman L.F., Blazer D.G., Scherr P.A., Wallace R.B. (1994). A Short Physical Performance Battery Assessing Lower Extremity Function: Association with Self-Reported Disability and Prediction of Mortality and Nursing Home Admission. J. Gerontol..

[42] Liver Frailty Index. https://liverfrailtyindex.ucsf.edu/.

[43] Wang M., Shui A.M., Barry F., Verna E., Kent D., Yao F., Seetharaman S., Berry K., Grubbs R.K., George G. (2023). The Tele-Liver Frailty Index (TeLeFI): Development of a Novel Frailty Tool in Patients with Cirrhosis via Telemedicine. Am. J. Transplant. Off. J. Am. Soc. Transplant. Am. Soc. Transpl. Surg..

[44] Lai J.C., Segev D.L., McCulloch C.E., Covinsky K.E., Dodge J.L., Feng S. (2018). Physical Frailty after Liver Transplantation. Am. J. Transplant. Off. J. Am. Soc. Transplant. Am. Soc. Transpl. Surg..

[45] Lai J.C., Covinsky K.E., McCulloch C.E., Feng S. (2018). The Liver Frailty Index Improves Mortality Prediction of the Subjective Clinician Assessment in Patients with Cirrhosis. Am. J. Gastroenterol..

[46] Xu C.Q., Yao F., Mohamad Y., Wong R., Kent D., Seetharaman S., Srisengfa Y., Lai J.C. (2021). Evaluating the Associations Between the Liver Frailty Index and Karnofsky Performance Status with Waitlist Mortality. Transplant. Direct.

[47] Lai J.C., Dodge J.L., Kappus M.R., Dunn M.A., Volk M.L., Duarte-Rojo A., Ganger D.R., Rahimi R.S., McCulloch C.E., Haugen C.E. (2020). Changes in Frailty Are Associated with Waitlist Mortality in Patients with Cirrhosis. J. Hepatol..

[48] Lai J.C., Shui A.M., Duarte-Rojo A., Ganger D.R., Rahimi R.S., Huang C.-Y., Yao F., Kappus M., Boyarsky B., McAdams-Demarco M. (2022). Frailty, Mortality, and Health Care Utilization after Liver Transplantation: From the Multicenter Functional Assessment in Liver Transplantation (FrAILT) Study. Hepatology.

[49] Lai J.C., Shui A.M., Duarte-Rojo A., Rahimi R.S., Ganger D.R., Verna E.C., Volk M.L., Kappus M., Ladner D.P., Boyarsky B. (2023). Association of Frailty with Health-Related Quality of Life in Liver Transplant Recipients. JAMA Surg..

[50] Clegg A., Rogers L., Young J. (2015). Diagnostic Test Accuracy of Simple Instruments for Identifying Frailty in Community-Dwelling Older People: A Systematic Review. Age Ageing.

[51] Sangro B., Argemi J., Ronot M., Paradis V., Meyer T., Mazzaferro V., Jepsen P., Golfieri R., Galle P., Dawson L. (2025). EASL Clinical Practice Guidelines on the Management of Hepatocellular Carcinoma. J. Hepatol..

[52] Singal A.G., Llovet J.M., Yarchoan M., Mehta N., Heimbach J.K., Dawson L.A., Jou J.H., Kulik L.M., Agopian V.G., Marrero J.A. (2023). AASLD Practice Guidance on Prevention, Diagnosis, and Treatment of Hepatocellular Carcinoma. Hepatology.

[53] Reig M., Forner A., Rimola J., Ferrer-Fàbrega J., Burrel M., Garcia-Criado Á., Kelley R.K., Galle P.R., Mazzaferro V., Salem R. (2022). BCLC Strategy for Prognosis Prediction and Treatment Recommendation: The 2022 Update. J. Hepatol..

[54] Ramai D., Dang-Ho K.P., Kewalramani A., Bandaru P., Sacco R., Giacomelli L., Shah A., Papa S., Cappellini F., Perversi F. (2021). Hospital Frailty Risk Score Is Independently Associated with Mortality and Encephalopathy in Hospitalized Patients with Hepatocellular Carcinoma. Biomedicines.

[55] Revoredo S., Del Fabbro E. (2023). Hepatocellular Carcinoma and Sarcopenia: A Narrative Review. Ann. Palliat. Med..

[56] Marasco G., Dajti E., Serenari M., Alemanni L.V., Ravaioli F., Ravaioli M., Vestito A., Vara G., Festi D., Golfieri R. (2022). Sarcopenia Predicts Major Complications after Resection for Primary Hepatocellular Carcinoma in Compensated Cirrhosis. Cancers.

[57] Hirota K., Kawaguchi T., Koya S., Nagamatsu A., Tomita M., Hashida R., Nakano D., Niizeki T., Matsuse H., Shiba N. (2020). Clinical Utility of the Liver Frailty Index for Predicting Muscle Atrophy in Chronic Liver Disease Patients with Hepatocellular Carcinoma. Hepatol. Res. Off. J. Jpn. Soc. Hepatol..

[58] Vitale A., Cabibbo G., Iavarone M., Viganò L., Pinato D.J., Ponziani F.R., Lai Q., Casadei-Gardini A., Celsa C., Galati G. (2023). Personalised Management of Patients with Hepatocellular Carcinoma: A Multiparametric Therapeutic Hierarchy Concept. Lancet Oncol..

[59] Vitale A., Trevisani F., Farinati F., Cillo U. (2020). Treatment of Hepatocellular Carcinoma in the Precision Medicine Era: From Treatment Stage Migration to Therapeutic Hierarchy. Hepatology.

[60] Masarone M., Cabibbo G., Pravisani R., Pelizzaro F., Torre P., Viganò M., Loglio A., Vitale A., Persico M. (2025). The Importance of Patient Fitness in Expert and Multidisciplinary Multiparametric Management of HCC: A Narrative Review. Hepatoma Res..

[61] DeMaria S., Khromava M., Schiano T.D., Lin H.-M., Kim S. (2019). Standardized Measures of Frailty Predict Hospital Length of Stay Following Orthotopic Liver Transplantation for Hepatocellular Carcinoma. Clin. Transplant..

[62] Yamada S., Shimada M., Morine Y., Imura S., Ikemoto T., Arakawa Y., Saito Y., Yoshikawa M., Miyazaki K. (2021). Significance of Frailty in Prognosis After Hepatectomy for Elderly Patients with Hepatocellular Carcinoma. Ann. Surg. Oncol..

[63] Tsuchihashi J., Koya S., Hirota K., Koga N., Narao H., Tomita M., Kawaguchi T., Hashida R., Nakano D., Tsutsumi T. (2021). Effects of In-Hospital Exercise on Frailty in Patients with Hepatocellular Carcinoma. Cancers.

[64] Okada T., Tanaka S., Shinkawa H., Ohira G., Kinoshita M., Amano R., Kimura K., Nishio K., Tauchi J., Uchida-Kobayashi S. (2024). Impact of Frailty on Long-Term Outcomes after Liver Resection for Hepatocellular Carcinoma in Elderly Patients: A Prospective Study. Asian J. Surg..

[65] Ozluk A.A., Williams G.R., Dai C., Goldberg J., Malla M., Pywell C., Siwakoti K., Outlaw D.A., Gupta G., El-Rayes B. (2024). Association between Frailty and Overall Survival among Older Adults with Hepatocellular Carcinoma. J. Geriatr. Oncol..

[66] Diao Y.-K., Li D., Wu H., Yang Y.-F., Wang N.-Y., Gu W.-M., Chen T.-H., Li J., Wang H., Zhou Y.-H. (2024). Association of Preoperative Frailty with Short- and Long-Term Outcomes after Hepatic Resection for Elderly Patients with Hepatocellular Carcinoma: Multicentre Analysis. BJS Open.

[67] Hu M.D., Chiou S.H., Delk M., Fenton C., Lai J.C., Li M. (2025). Physical Function as Assessed by the Liver Frailty Index Remains Stable Over Time in Patients with Hepatocellular Carcinoma Treated with Locoregional Therapies. Clin. Transl. Gastroenterol..

[68] Shao Y.-F., Zu Y.-N., Yin X.-Q., Xiao J.-C., Gu Y.-M. (2025). Impact of Frailty on the Long-Term Prognosis of the Elderly with Hepatocellular Carcinoma Treated with Transarterial Chemoembolization. Sci. Rep..

[69] Jutras G., Far A., Wang M., Li M., Ge J., Lai J.C. (2025). Tumor Burden and Frailty: No Association in Liver Transplant Candidates with Hepatocellular Carcinoma. Liver Transplant. Off. Publ. Am. Assoc. Study Liver Dis. Int. Liver Transplant. Soc..

[70] Velanovich V., Antoine H., Swartz A., Peters D., Rubinfeld I. (2013). Accumulating Deficits Model of Frailty and Postoperative Mortality and Morbidity: Its Application to a National Database. J. Surg. Res..

[71] Sandini M., Pinotti E., Persico I., Picone D., Bellelli G., Gianotti L. (2017). Systematic Review and Meta-Analysis of Frailty as a Predictor of Morbidity and Mortality after Major Abdominal Surgery. BJS Open.

[72] Hall D.E., Arya S., Schmid K.K., Carlson M.A., Lavedan P., Bailey T.L., Purviance G., Bockman T., Lynch T.G., Johanning J.M. (2017). Association of a Frailty Screening Initiative with Postoperative Survival at 30, 180, and 365 Days. JAMA Surg..

[73] Haugen C.E., McAdams-DeMarco M., Holscher C.M., Ying H., Gurakar A.O., Garonzik-Wang J., Cameron A.M., Segev D.L., Lai J.C. (2020). Multicenter Study of Age, Frailty, and Waitlist Mortality Among Liver Transplant Candidates. Ann. Surg..

[74] Eochagain C.M., Neuendorff N.R., Gente K., Leipe J., Verhaert M., Sam C., de Glas N., Kadambi S., Canin B., Gomes F. (2025). Management of Immune Checkpoint Inhibitor-Associated Toxicities in Older Adults with Cancer: Recommendations from the International Society of Geriatric Oncology (SIOG). Lancet Oncol..

[75] Eng L., Sutradhar R., Kaliwal Y., Niu Y., Liu N., Liu Y., Powis M.L., Liu G., Peppercorn J.M., Krzyzanowska M.K. (2022). Impact of Age and Frailty on Acute Care Use during Immune Checkpoint Inhibitor (ICI) Treatment: A Population-Based Study. J. Clin. Oncol..

[76] Jiménez Galán R., Prado-Mel E., Alvarez de Sotomayor M., Martin L.A.-K. (2023). Impact of Frailty on Outcomes of First-Line Pembrolizumab Monotherapy in a Real-World Population with Advanced Non-Small Cell Lung Cancer. Biology.

[77] Lai J.C., Tandon P., Bernal W., Tapper E.B., Ekong U., Dasarathy S., Carey E.J. (2021). Malnutrition, Frailty, and Sarcopenia in Patients with Cirrhosis: 2021 Practice Guidance by the American Association for the Study of Liver Diseases. Hepatology.

[78] Yang L., Courneya K.S., Friedenreich C.M. (2024). The Physical Activity and Cancer Control (PACC) Framework: Update on the Evidence, Guidelines, and Future Research Priorities. Br. J. Cancer.

[79] Stout N.L., Mina D.S., Lyons K.D., Robb K., Silver J.K. (2021). A Systematic Review of Rehabilitation and Exercise Recommendations in Oncology Guidelines. CA Cancer J. Clin..

[80] Christopher C.N., Kang D.-W., Wilson R.L., Gonzalo-Encabo P., Ficarra S., Heislein D., Dieli-Conwright C.M. (2023). Exercise and Nutrition Interventions for Prehabilitation in Hepato-Pancreato-Biliary Cancers: A Narrative Review. Nutrients.

